# *PTEN* Methylation Promotes Inflammation and Activation of Fibroblast-Like Synoviocytes in Rheumatoid Arthritis

**DOI:** 10.3389/fphar.2021.700373

**Published:** 2021-07-08

**Authors:** Xiao-Feng Li, Sha Wu, Qi Yan, Yuan-Yuan Wu, He Chen, Su-Qin Yin, Xin Chen, Hua Wang, Jun Li

**Affiliations:** ^1^ Inflammation and Immune Mediated Disease Laboratory of Anhui Province, School of Pharmacy, Anhui Institute of Innovative Drugs, Anhui Medical University, Hefei, China; ^2^ The Key Laboratory of Anti-Inflammatory and Immune Medicines, Ministry of Education, Hefei, China; ^3^ Postdoctoral Station of Clinical Medicine of Anhui Medical University, Hefei, China; ^4^ Department of Biochemistry and Molecular Biology, School of Basic Medical Sciences, Anhui Medical University, Hefei, China; ^5^ Departments of Clinical Laboratory, the First Affiliated Hospital of Anhui Medical University, Hefei, China; ^6^ Department of Oncology, the First Affiliated Hospital of Anhui Medical University, Hefei, China

**Keywords:** rheumatoid arthritis, pten, inflammation, DNA methylation, fibroblast-like synoviocytes

## Abstract

Rheumatoid arthritis (RA) is characterized by a tumor-like expansion of the synovium and subsequent destruction of adjacent articular cartilage and bone. In our previous work we showed that phosphatase and tension homolog deleted on chromosome 10 (*PTEN*) contributes to the activation of fibroblast-like synoviocytes (FLS) in adjuvant-induced arthritis (AIA), but the underlying mechanism is not unknown. In this study, we show that *PTEN* is downregulated while DNA methyltransferase (DNMT)1 is upregulated in FLS from RA patients and a rat model of AIA. DNA methylation of *PTEN* was increased by administration of tumor necrosis factor (TNF)-α in FLS of RA patients, as determined by chromatin immunoprecipitation and methylation-specific PCR. Treatment with the methylation inhibitor 5-azacytidine suppressed cytokine and chemokine release and FLS activation *in vitro* and alleviated paw swelling *in vivo*. *PTEN* overexpression reduced inflammation and activation of FLS *via* protein kinase B (AKT) signaling in RA, and intra-articular injection of PTEN-expressing adenovirus into the knee of AIA rats markedly reduced inflammation and paw swelling. Thus, *PTEN* methylation promotes the inflammation and activation of FLS in the pathogenesis of RA. These findings provide insight into the molecular basis of articular cartilage destruction in RA, and indicate that therapeutic strategies that prevent *PTEN* methylation may an effective treatment.

## Introduction

Rheumatoid arthritis (RA) is a chronic and systemic autoimmune disease characterized by inflammation and hyperplasia of synovial tissues and destruction of adjacent articular cartilage (Smolen et al., 2018). Fibroblast-like synoviocytes (FLS), the major cell type in synovial tissue, are involved in the pathologic and inflammatory processes of RA (De Oliveira et al., 2019). Activated FLS secrete proinflammatory cytokines (tumor necrosis factor (TNF)-α and interleukin [IL]-1β), chemokines [monocyte chemoattractant protein (MCP)-1, also known as C–C motif chemokine ligand (CCL)-2], matrix metalloproteinases [(MMP)-3 and -9], and angiogenic factors (Yoshitomi, 2019) that enter the intra-articular synovial fluid and destroy cartilage and bone in RA (Ai et al., 2018). In fact, FLS activation and immune dysregulation are the main factors contributing to the pathogenesis and development of RA (Yoshitomi, 2019). Therefore, inhibiting FLS activation and inflammation is important for the treatment of RA.

Crosstalk between cytokine and chemokine signaling contributes to RA progression by activating FLS (Muntyanu et al., 2016). The proinflammatory cytokines TNF-α and IL-6 are known to be involved in this process (Tai et al., 2021); therapeutic strategies that restore their levels could prevent RA development. TNF-α is present at a high concentration in the serum and synovial fluid of RA patients, and exposure of FLS from these patient to high levels of TNF-α decreased total histone levels and increased acetylation of the remaining histones through recruitment of nuclear factor (NF)-κB p65 (Sohn et al., 2015). Chemokines exacerbate joint inflammation by recruiting inflammatory cells to the synovial microenvironment. Macrophage inhibitory protein (MIP)-1α (also known as CCL-3) and CCL-2 were shown to participate in macrophage activation in the synovium of RA patients (Kabala et al., 2020).

We recently reported that phosphatase and tension homolog deleted on chromosome 10 (PTEN) is involved in the activation and inflammation of FLS in RA (Li et al., 2019a). PTEN was shown to exert anti-inflammatory and antiproliferative effects by inhibiting the activation of protein kinase B (AKT) signaling. Additionally, adenovirus-mediated delivery of *PTEN* reduced articular index, ankle circumference, and histology scores and decreased vascular endothelial growth factor (VEGF) and IL-1β levels in collagen-induced arthritis (Wang et al., 2008). Our previous research showed that *PTEN* overexpression suppressed the proliferation and migration of FLS and inflammation in adjuvant-induced arthritis (AIA) (Li et al., 2019a), which may be regulated by DNA methylation (Li et al., 2019a). However, the precise mechanism underlying the aberrant expression of *PTEN* in RA and its relationship to inflammation and FLS activation are unknown. This was addressed in the present study both *in vitro* using FLS from patients with RA as well as *in vivo* using a rat model of AIA.

## Methods and Methods

### FLS Isolation

FLS were extracted from the synovium of patients with RA (*n* = 6) or osteoarthritis (OA; *n* = 8) undergoing total joint replacement at the Department of Orthopedics, the First Affiliated Hospital of Anhui Medical University, Hefei, China. All patients provided written, informed consent for the use of their samples, and experimental protocols involving human subjects were approved by the biomedical ethics committee of Anhui Medical University (approval no. 20200970). Cells between passages 4 and 9 were used for experiments. The information of RA patients are shown in Supplementary Table S1.

### Rat Model of AIA, *PTEN* Overexpression, and DNA Methylation Inhibitor Treatment

To establish the rat AIA model, Sprague–Dawley rats (80–120 g) were treated with complete Freund’s adjuvant (0.1 ml/100 g body weight; Chondrex, Redmond, WA, United States) for 24 days *via* subcutaneous injection into the left hind paw (Li et al., 2017; Li X.-F. et al., 2019; Li XF. et al., 2019). Control rats were injected with saline. After 14 days, AIA rats were administered adenovirus carrying the rat *PTEN* gene (Ad-PTEN) or the green fluorescent protein gene (Ad-GFP) (Hanbio, Shanghai, China) *via* intra-articular injection (0.1 ml) into the hind knee. They were also treated with the DNA hypomethylating agent 5-aza-2′-deoxycytidine (5-azadC; Sigma-Aldrich, St. Louis, MO; United States) by intraperitoneal injection at a dose of 0.7 mg/kg/3 days for 21 days. The rats were provided by the Experimental Animal Center of Anhui Medical University and were maintained in accordance with the Guides for the Care and Use of Laboratory Animals of the Center for Developmental Biology, Anhui Medical University. Experiments involving the animals were carried out in accordance with the Regulations of Experimental Animal Administration issued by the State Committee of Science and Technology of China, and were approved by Anhui Medical University’s subcommittee on animal care (approval no. 20200963).

### Histopathology

Synovium specimens from human and rat knee joints were fixed with 4% paraformaldehyde for 48 h and embedded in paraffin according to standard procedures, and sectioned for hematoxylin and eosin staining, immunohistochemistry, and immunofluorescence analysis. The sections were photographed using CaseViewer software (3DHISTECH, Budapest, Hungary).

### 2.4 Enzyme-Linked Immunosorbent Assay (ELISA)

Rat blood was collected through the abdominal aorta and the serum was separated by centrifugation. TNF-α and IL-6 levels in rat serum and IL-6 and IL-8 levels in human FLS from RA patients were measured by ELISA using commercial kits (R&D, Minneapolis, MN, United States) according to the manufacturer’s protocol.

### FLS Culture

FLS were obtained from the synovium of AIA rats and RA patients by tissue separation and cultured in Dulbecco’s Modified Eagle’s Medium (HyClone, South Logan, UT, United States) supplemented with 20% (v/v) fetal bovine serum, 100 U/ml penicillin, and 100 mg/ml of streptomycin (Beyotime, Shanghai, China) at 37°C and 5% CO_2_.

### Immunocytochemistry

PTEN expression in FLS treated with TNF-α was detected by immunocytochemistry using a rabbit anti-PTEN antibody (Abcam, Cambridge, United Kingdom). The cells were stained with 4′,6-diamidino-2-phenylindole (Beyotime, Shanghai, China) in the dark and then photographed under an epifluorescence microscope (BX-51; Olympus, Tokyo, Japan).

### Gene Silencing by RNA Interference (RNAi)

FLS were transfected with *PTEN* small interfering RNA (GenePharma, Shanghai, China) or overexpression plasmid (GeneChem, Shanghai, China) using Lipofectamine 2000 (Invitrogen, Carlsbad, CA, United States). The sense and antisense oligonucleotide sequences were as follows: human PTEN-RNAi, 5′-CAG​UAG​AGG​AGC​CGU​CAA​ATT-3′ and 5′-UUU​GAC​GGC​UCC​UCU​ACU​GTT-3′. A negative control scrambled RNAi was used in parallel. FLS were transfected with PTEN-pcDNA3.1 (human) vectors to overexpress PTEN, and with empty pcDNA 3.1 vector as a control. After transfection for 8 h, FLS were cultured in complete medium at 37°C for 48 h before analysis.

### Adenovirus-Mediated *PTEN* Overexpression in FLS

Ad-PTEN and Ad-GFP (negative control) were prepared as stock solutions of 1 × 10^10^ PFU/ml were obtained from Hanbio (Shanghai) and used to infect the synovium of AIA rats.

### Methylation-specific PCR

DNA was extracted from FLS with the Wizard DNA Clean-Up System (Promega, Madison, WI, United States) according to the manufacturer’s instructions. Unmethylated cytosine residues were converted to uracil with the Methylamp DNA Modification Kit (EpiGentek, Farmingdale, NY, United States). The primer sequences for amplification of methylated and unmethylated *PTEN* are shown in Supplementary Table S2.

### Quantitative Real-Time PCR

Total RNA was extracted from FLS using TRIzol reagent (Invitrogen) and reverse transcribed to cDNA with the iScript cDNA kit (Bio-Rad, Hercules, CA, United States). qRT-PCR was performed with SYBR Green q-PCR Master Mix (Toyobo, Osaka, Japan). Primers for qRT-PCR were synthesized by Sangon Biotech (Shanghai, China), and the sequences are shown in Supplementary Table S3. All reactions were performed three times, and relative mRNA expression levels of target genes were determined by normalization to the level of β-actin.

### Western Blotting

Total protein was extracted from FLS with lysis buffer. The proteins were denatured by boiling and separated by 10% sodium dodecyl sulfate polyacrylamide gel electrophoresis and transferred to a polyvinylidene difluoride membrane (Millipore, Bedford, MA, United States) that was blocked and incubated overnight with primary antibodies. Rabbit antibodies PTEN; tissue inhibitor of metalloproteinase (TIMP)-1 (Abcam); AKT, phosphorylated (p-)AKT, MMP-3, and MMP-9 (Cell Signaling Technology, Danvers, MA, United States); and IL-1β, IL-6, and IL-17A, (Bioworld, Shanghai, China) were used at 1:500 dilution. Mouse anti-DNA methyltransferase (DNMT)1 (Abcam) and anti-β-actin (Bioworld) antibodies were used at 1:1,000 dilution. After washing, the membrane was incubated with horseradish peroxidase (HRP)-conjugated goat anti-mouse or -rabbit antibodies for 1 h. Protein bands were visualized with Immobilon Western Chemiluminescent HRP Substrate (Millipore) and photographed with a ChemiDoc^MP^ Imaging System (Bio-Rad).

### Chromatin Immunoprecipitation

ChIP was performed using the SimpleChIP Kit (Cell Signaling Technology) according to the manufacturer’s instructions. Proteins in FLS were crosslinked with 1% formaldehyde and the cells were lysed; the lysate was incubated overnight at 4°C with anti-DNMT1 mouse monoclonal antibody followed by ChIP-grade Protein A/G Plus agarose beads for 2 h. After washing with three different buffers, the crosslinks were reversed at 65°C for 2 h, and immunoprecipitated DNA was detected by qRT-PCR.

### Paw Swelling

After AIA rats were treated with complete Freund’s adjuvant subcutaneous injection into the left hind paw, the volume of right hind paw swelling was measured by toe volume measuring instrument (Chengdu Taimeng Software Co. Ltd.) at days 15, 18, 21, 24, 27, and 30.

### Statistical Analysis

Data are presented as mean ± standard deviation and were analyzed using SPSS v13.0 software. Statistical significance was determined by 1-way analysis of variance or RMANOVA with a post-hoc Dunnett’s test. In all analyses, *p* values < 0.05 were considered statistically significant.

## Results

### *PTEN* Expression is Downregulated in FLS From Both Clinical RA Specimens and AIA Rats

**FIGURE 1:**
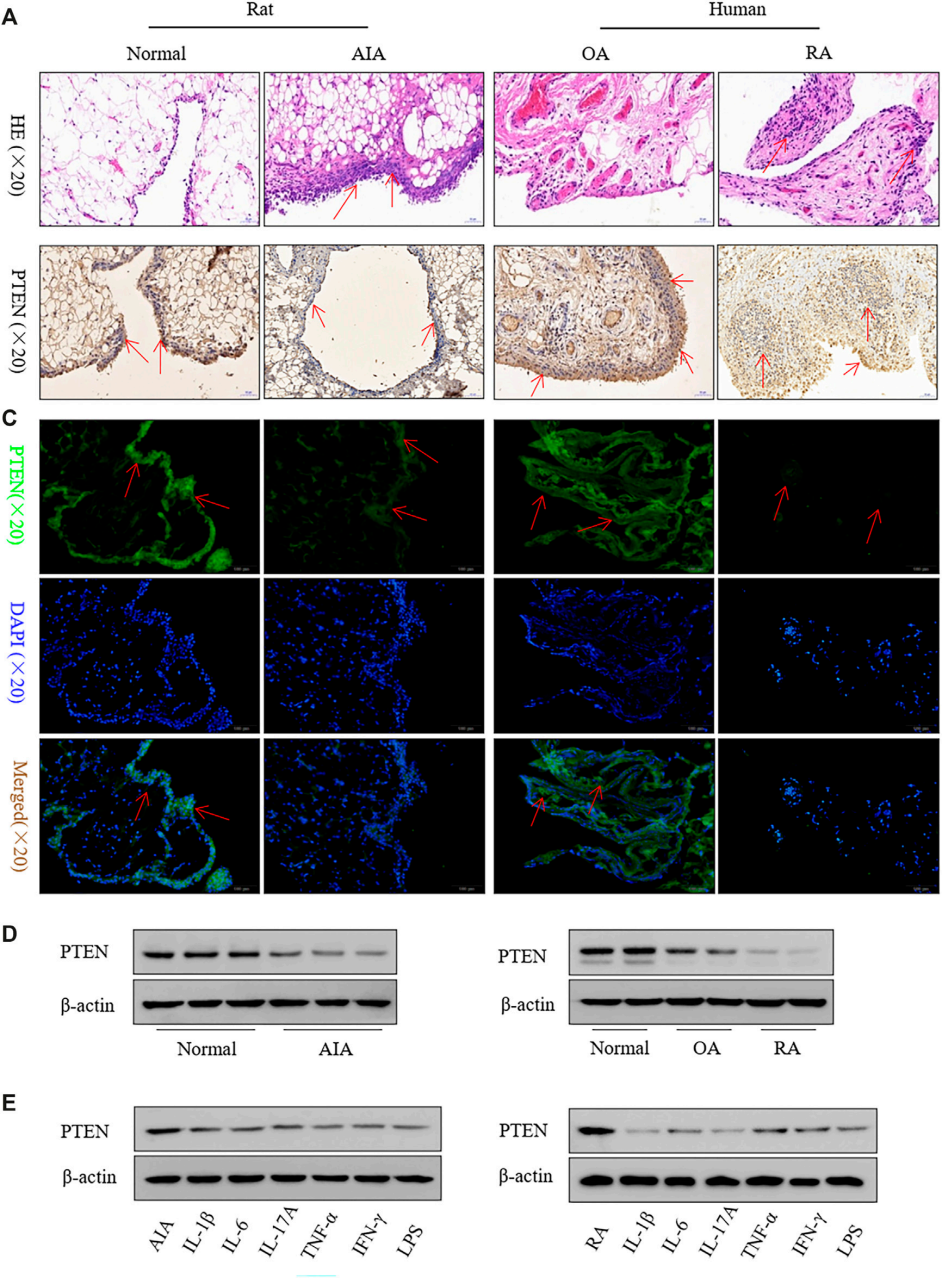
PTEN expression is reduced in FLS from clinical RA specimens and a rat model of AIA. **(A)** Representative images of hematoxylin and eosin staining in synovium. **(B, C)** Representative images of PTEN immunoreactivity in synovium detected by immunohistochemistry **(B)** and immunofluorescence labeling **(C)**. **(D)** Western blot analysis of PTEN protein levels. **(E)** and after treatment with IL-1β (2 ng/ml), IL-6 (5 ng/ml), IL-17A (10 ng/ml), TNF-α (10 ng/ml), IFN-γ (10 ng/ml), and lipopolysaccharide (LPS; 1 μg/ml). Values represent mean ± SD of three different FLS samples.

Inflammatory cell infiltration into synovial tissue was observed in rat AIA and clinical RA specimens (Figure 1A). PTEN was downregulated in the lining and in the sublining of the synovium by immunohistochemistry (Figure 1B) and immunofluorescence analysis (Figure 1C), which was confirmed by western blotting (Figure 1D). Treatment of FLS with inflammatory factors including IL-1β, IL-6, IL-17A, TNF-α, IFN-γ, and lipopolysaccharide reduced PTEN levels (Figure 1E).

### *PTEN* Expression in RA Is Regulated by DNA Methylation

**FIGURE 2:**
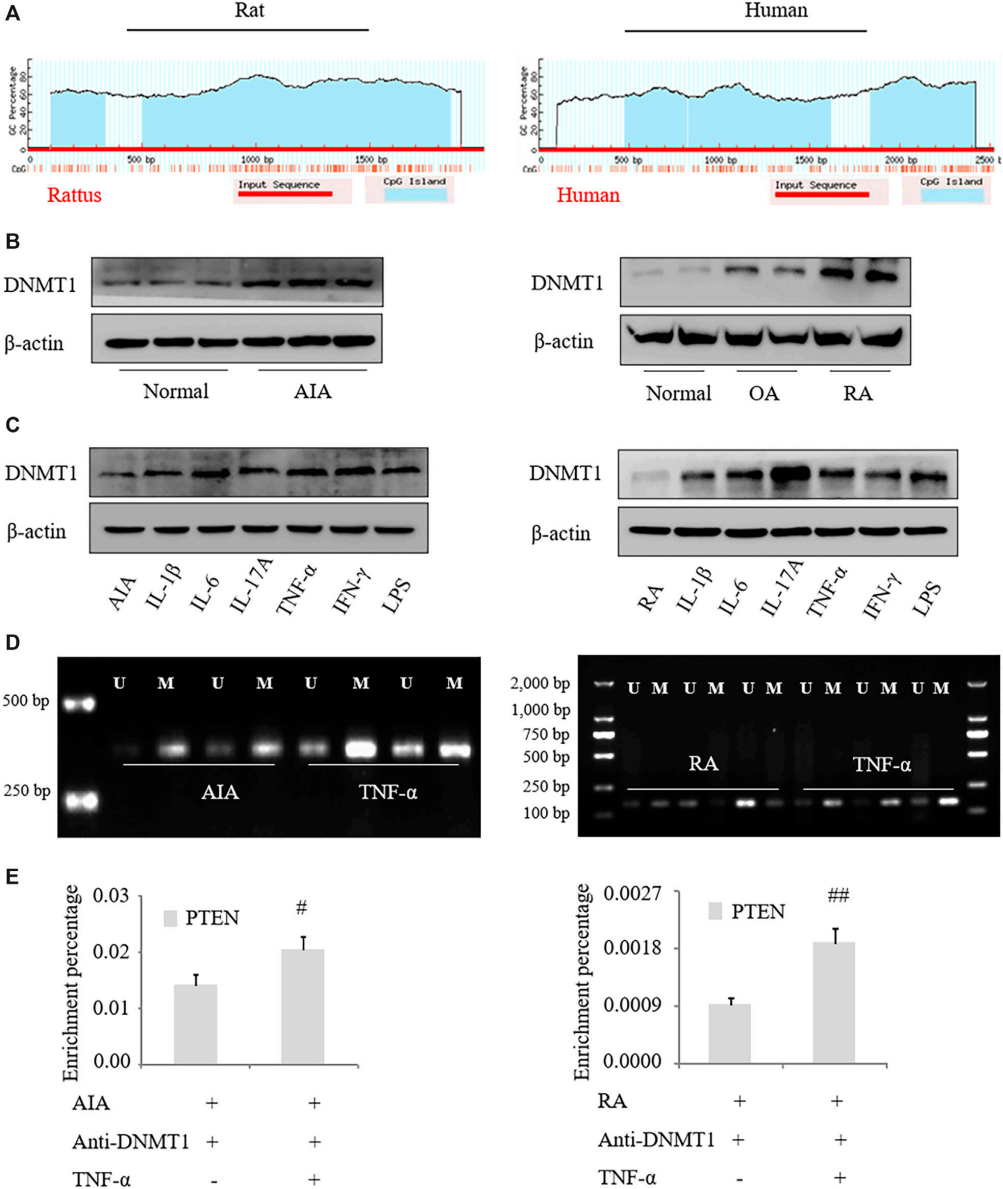
***PTEN*** expression is regulated by DNA methylation in RA. **(A)** Schematic illustration of rat and human *PTEN* genes; a CpG island was detected near and upstream of the first exon. **(B, C)** DNMT1 protein expression in FLS from RA patients and AIA model rats was analyzed by western blotting. **(D)** and after treatment with IL-1β (2 ng/ml), IL-6 (5 ng/ml), IL-17A (10 ng/ml), TNF-α (10 ng/ml), IFN-γ (10 ng/ml), and lipopolysaccharide (LPS; 1 μg/ml). **(D)** MSP analysis of *PTEN* methylation level in FLS treated with TNF-α. **(E)** ChIP of *PTEN* mRNA precipitated with anti-DNMT1 antibody from FLS treated with TNF-α. Values represent mean ± SD. ^#^ *p* < 0.05, ^##^ *p* < 0.01 vs. AIA group or RA group.

We identified CpG islands near the first exon of the *PTEN* transcript and in the upstream region of the human and rat genes, suggesting that the change in *PTEN* expression in RA is related to CpG methylation (Figure 2A). DNMT1 protein was upregulated in FLS from RA patients compared to those from OA patients (Figure 2B) and in FLS from AIA rats compared to those from normal rats. DNMT1 expression was also increased to varying degrees in FLS treated with proinflammatory factors (Figure 2C). TNF-α treatment resulted in methylation of the *PTEN* gene, as detected by the MSP assay (Figure 2D), and recruited DNMT1 to the coding region of the *PTEN* gene, as determined by ChIP (Figure 2E). These results indicate that the *PTEN* gene is downregulated by DNA methylation in RA.

### Inhibiting DNA Methylation Suppresses Inflammation and Activation of FLS in RA

**FIGURE 3:**
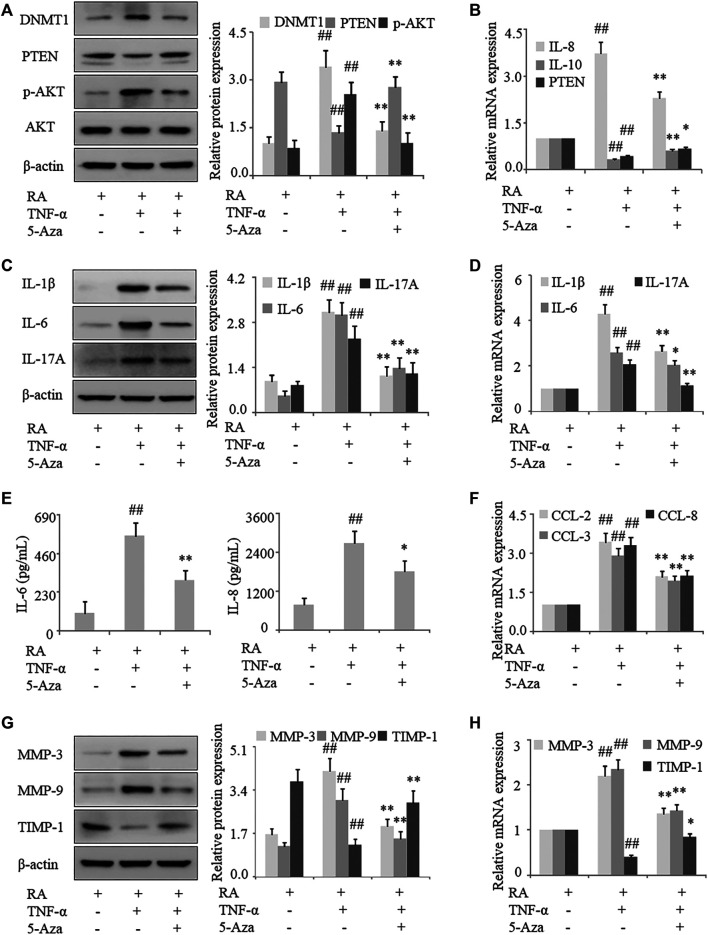
Inhibition of DNA methylation suppresses inflammation and FLS activation in RA. FLS from RA patients were treated with TNF-α (10 ng/ml) and 5-azadC (2 μM). **(A)** DNMT1, PTEN, and *p*-AKT protein levels detected by western blotting. **(B)** *PTEN*, *IL-10*, and *IL-8* mRNA levels detected by qRT-PCR. **(C, D)** IL-1β, IL-6, and IL-17A mRNA and protein levels analyzed by qRT-PCR and western blotting, respectively. **(E)** IL-6 and IL-8 in the culture supernatant detected by ELISA. **(F)** *CCL-2*, *CCL-3*, and *CCL-8* mRNA levels detected by qRT-PCR. **(G, H)** MMP-3, MMP-9, and TIMP-1 mRNA and protein levels analyzed by qRT-PCR and western blotting, respectively. Values represent mean ± SD. ^##^ *p* < 0.01 vs. AIA or RA group; *p < 0.05, **p < 0.01 vs. TNF-α group.

To confirm that *PTEN* expression in RA is regulated by DNA methylation, human RA FLS were treated with the methylation inhibitor 5-azadC; this resulted in the upregulation of PTEN (Figures 3A,B) and downregulation of DNMT1 protein treated with TNF-α. Notably, following treatment with TNF-α, expression of the proinflammatory cytokines IL-1β, IL-6, and IL-17A in FLS were decreased by 5-azadC, as determined by western blotting and qRT-PCR (Figures 3C,D), while secretion of IL-6 and IL-8 was also reduced (Figure 3E) along with the mRNA expression of the chemokines CCL-2, CCL-3, CCL-8, IL-8, and IL-10 (Figures 3B,F) and MMP-3 and MMP-9 (Figures 3G,H). Thus, inhibiting the methylation of *PTEN* results in its upregulation and decreases proinflammatory cytokine and chemokine levels and FLS activation in RA.

**FIGURE 4:**
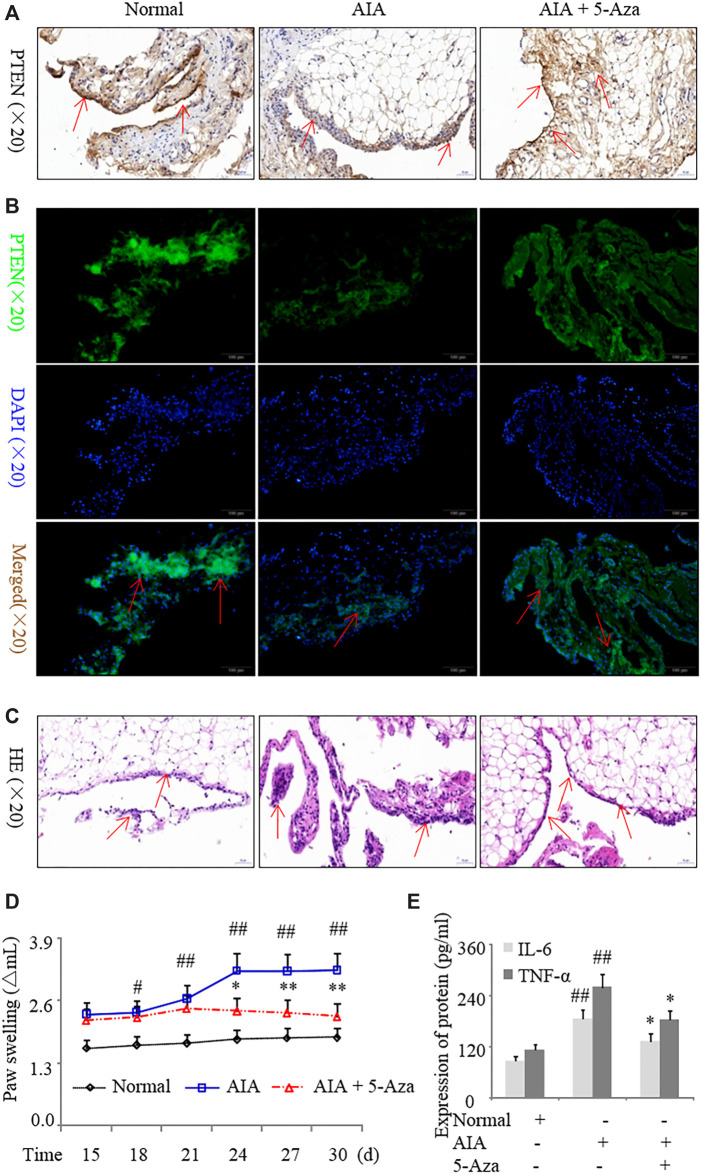
Inhibition of DNA methylation suppresses inflammation in a rat model of AIA. AIA rats were treated with 5-azadC (0.7 mg/kg) by intraperitoneal injection. **(A, B)** Representative images of PTEN immunoreactivity in the synovium detected by immunohistochemistry **(A)** and immunofluorescence labeling **(B)**. **(C)** Representative images of hematoxylin and eosin staining in the synovium. **(D)** Paw swelling. **(E)** Serum IL-6 and TNF-α protein levels detected by ELISA. Values represent mean ± SD. ^#^ *p* < 0.05, ^##^ *p* < 0.01 vs. normal group; *p < 0.05, **p < 0.01 vs. AIA group.

Intraperitoneal injection of 5-azadC into the synovium of AIA rats caused an increase in PTEN expression compared to model rats without treatment (Figures 4A,B); this was accompanied by decreased inflammatory cell infiltration (Figure 4C) and paw swelling (Figure 4D); reduced serum levels of IL-6 and TNF-α protein (Figure 4E); and downregulation of IL-1β, IL-17A, and DNMT1 and upregulation of PTEN in synovial tissues (Supplementary Figures 1A–D). These results indicate that inhibiting DNMT1 activity reduces *PTEN* methylation and suppresses inflammation in RA.

### *PTEN* Overexpression Inhibits Inflammation and Activation of FLS in RA

**FIGURE 5:**
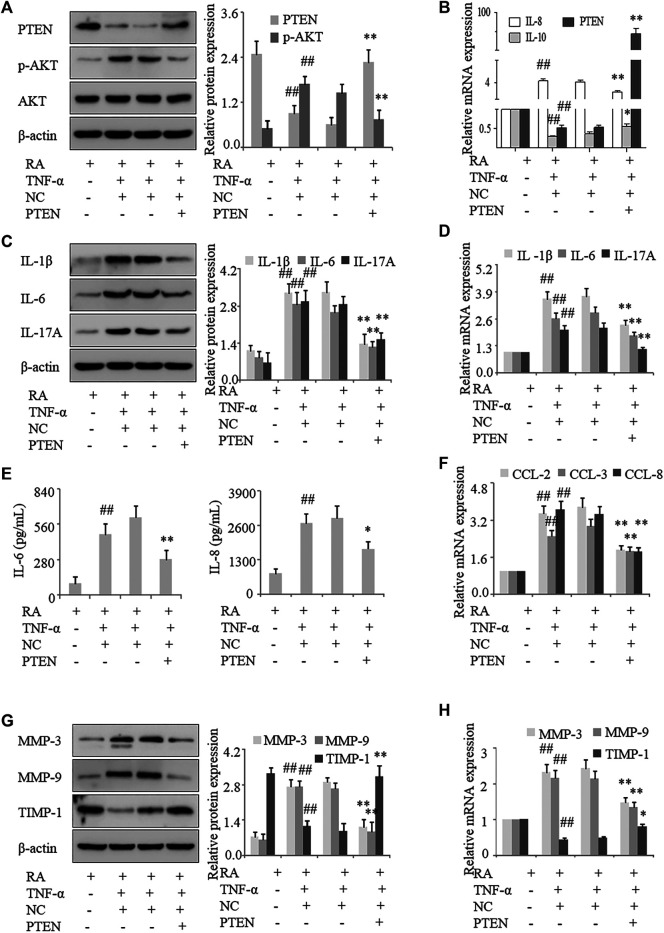
PTEN overexpression inhibits inflammation and FLS activation in RA. FLS from the synovium of RA patients overexpressing PTEN were treated with TNF-α (10 ng/ml). **(A)** PTEN and *p*-AKT protein levels detected by western blotting. **(B)** *PTEN*, *IL-10*, and *IL-8* mRNA levels detected by qRT-PCR. **(C, D)** IL-1β, IL-6, and IL-17A mRNA and protein levels analyzed by qRT-PCR and western blotting, respectively. **(E)** IL-6 and IL-8 protein levels in the culture supernatant detected by ELISA. **(F)** *CCL-2*, *CCL-3*, and *CCL-8* mRNA levels detected by qRT-PCR. **(G, H)** MMP-3, MMP-9, and TIMP-1 protein levels detected by western blotting. Values represent mean ± SD. ^##^ *p* < 0.01 vs. RA group; *p < 0.05, **p < 0.01 vs. pcDNA3.1 group.

To clarify the mechanism underlying the regulation of inflammation by PTEN in RA, we used the human PTEN-pcDNA3.1 vector to overexpress *PTEN* in FLS. Western blot and qRT-PCR analyses revealed that PTEN expression was increased in cells overexpressing *PTEN*, which was accompanied by upregulation of *p*-AKT (Figures 5A,B). In human RA FLS treated with TNF-α, the levels of the proinflammatory cytokines IL-1β, IL-6, IL-8, and IL-17A were decreased (Figures 5C,D) whereas IL-10 mRNA level was increased (Figure 5B) by PTEN overexpression; moreover, IL-6 and IL-8 secretion was reduced (Figure 5E) along with the mRNA and protein expression of CCL-2, CCL-3, and CCL-8 (Figure 5F) and MMP-3 and MMP-9, with TIPM-1 levels showing the opposite trends (Figures 5E,H). These results provide further evidence that decreased expression of PTEN leads to increased inflammation and FLS activation in RA.

**FIGURE 6:**
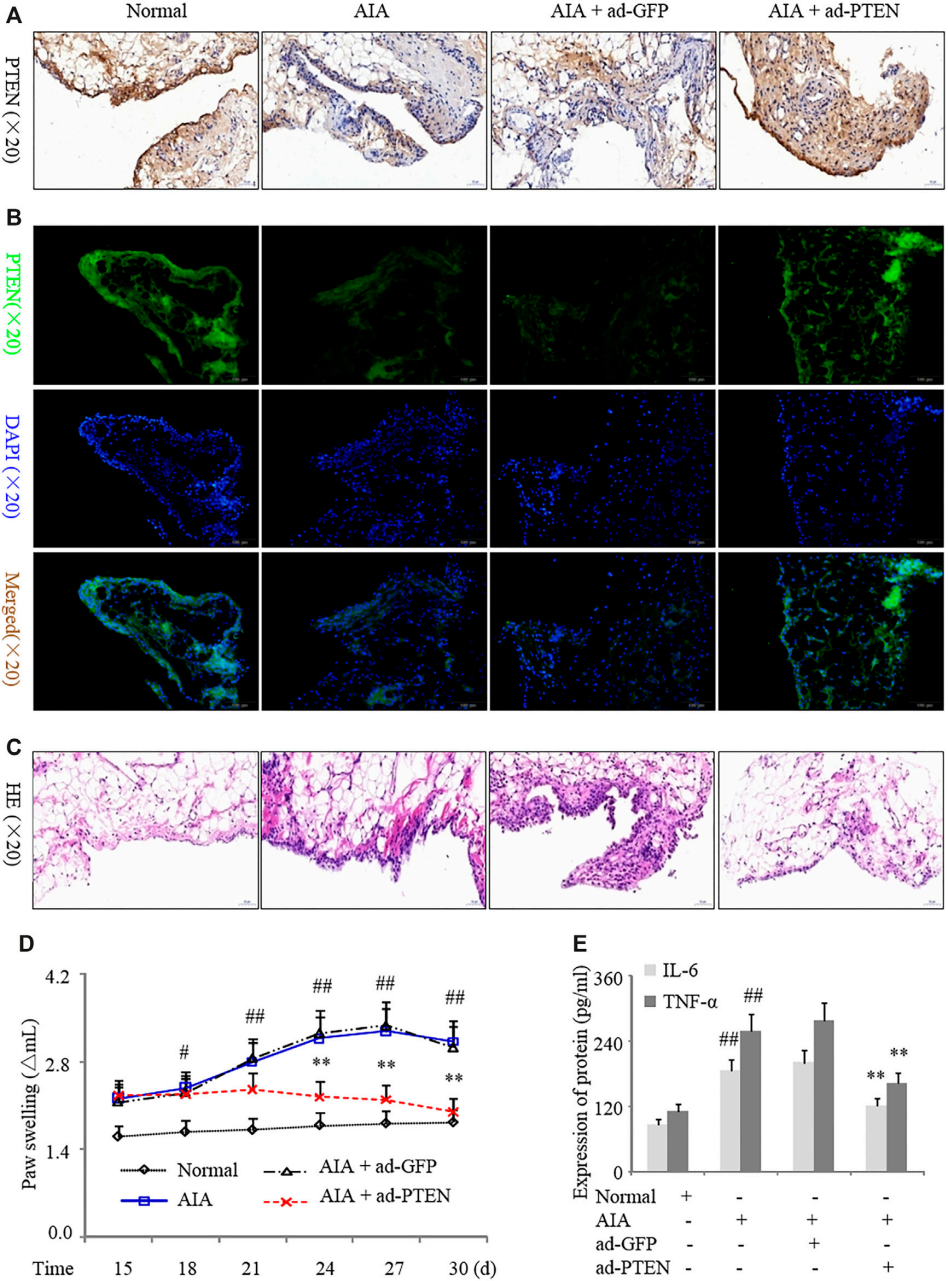
PTEN overexpression suppresses inflammation in a rat AIA model. AIA model rats were treated with Ad-GFP or Ad-PTEN by intra-articular injection. **(A, B)** Representative images of PTEN immunoreactivity in synovium detected by immunohistochemistry **(A)** and immunofluorescence analysis **(B)**. **(C)** Representative images of hematoxylin and eosin staining in the synovium. **(D)** Paw swelling. **(E)** Serum IL-6 and TNF-α protein levels detected by ELISA. Values are expressed as mean ± SD. ^#^ *p* < 0.05, ^##^ *p* < 0.01 vs. normal group; *p < 0.05, **p < 0.01 vs. Ad-GFP group.

In order to assess the effect of PTEN overexpression in synovial tissues *in vivo*, we injected Ad-PTEN or the control adenovirus Ad-GFP into the knees of AIA rats. We confirmed that Ad-PTEN caused the upregulation of PTEN in the synovial lining compared to Ad-GFP by western blotting (Figures 6A,B). Intra-articular injection of Ad-PTEN decreased inflammatory cell infiltration into the synovium (Figure 6C), with a corresponding reduction in paw swelling (Figure 6D). Moreover, serum IL-6 and TNF-α levels were also downregulated (Figure 6E) along with IL-1β, IL-6, IL-17A, and TNF-α levels in synovial tissues (Supplementary Figures 1E, F) in AIA rats injected with Ad-PTEN as compared to Ad-GFP. Thus, overexpressing PTEN in the synovial joint of RA model rats inhibits the expression of proinflammatory cytokines and reduces paw swelling.

## Discussion

**FIGURE 7:**
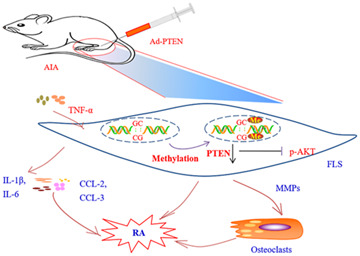
*PTEN* methylation promotes inflammation and FLS activation in RA. Methylation-induced downregulation of *PTEN* promotes inflammation and activation of FLS in the development of RA *via* the proinflammatory factors IL-1β, IL-6, CCL-2, and CCL3. TNF-α produced by inflammatory cells (eg., macrophages and T cells) induces the activation of FLS and aggravates cartilage destruction. Overexpression of PTEN reversed these pathologic effects.

RA is characterized by interactions between FLS and inflammatory cells in the synovium. *PTEN* is downregulated in RA, which was shown to be correlated with joint inflammation in AIA rats. However, the role of PTEN in the pathogenesis of RA is not fully understood. In the current study, we confirmed that *PTEN* was downregulated whereas *DNMT1* was upregulated in human RA and rat AIA FLS. We also found that methylation of the *PTEN* gene was increased in RA FLS treated with TNF-α by ChIP and with the MSP assay. Administration of the DNA methylation inhibitor 5-azadC suppressed inflammation, FLS activation, and paw swelling in AIA rats; *PTEN* was found to mediate this effect *via* AKT signaling. Accordingly, intra-articular injection of Ad-PTEN into the knees of AIA rats markedly reduced inflammation and paw swelling. We also demonstrated that *PTEN* methylation promotes inflammation (as evidenced by the upregulation of IL-1β, IL-6, CCL-2, and CCL-3) and activation of FLS in RA (Figure 7).

The rat AIA model has histologic and immunologic features similar to RA in humans and is a widely used model in RA research (Du et al., 2019). The pathogenesis of RA involves the activation of various inflammatory cell types including innate immune cells (e.g., mast cells and macrophages) (Andreev et al., 2020), adaptive immune cells (T and B cells) (Clarke, 2020), and FLS that interact to modulate the immune response. Activated FLS promote the infiltration, recruitment, and accumulation of T lymphocytes and macrophages in RA by producing proinflammatory cytokines, extracellular matrix proteins, chemokines, and cell adhesion molecules. However, to date there are no therapeutics that effectively target FLS, because the mechanism regulating FLS activation is not known. Inhibiting the production of proinflammatory cytokines and chemokines by FLS is a potential strategy for RA treatment.

*PTEN* mRNA expression is regulated by DNA methylation in gastric cancer (Zhang et al., 2019) and melanoma (Roh et al., 2016; Giles et al., 2019). DNA methylation is a type of epigenetic modification that regulates gene expression and may contribute to immune dysregulation (Zheng et al., 2010; Ai et al., 2018). Methylation of the poly (ADP-ribose) polymerase family member (PARP)9 gene was correlated with the level of transcript in Jurkat cells and T lymphocytes isolated from patients with RA (Zhu et al., 2019). We previously reported that PTEN expression was undetectable in the synovial lining of RA patients (Pap et al., 2000; Chen et al., 2017); in the present work, we observed PTEN expression in RA synovial tissue following treatment with inflammatory factors *in vitro* and *in vivo*. We also provide the first detailed evidence that PTEN regulates FLS activation and production of proinflammatory cytokines and chemokines in RA pathogenesis, which was dependent on *PTEN* methylation status.

In the present study, DNMT1 was upregulated in FLS from clinical RA specimens and a rat model of AIA that were treated with TNF-α, which was accompanied by DNMT1 recruitment to the coding region of the *PTEN* gene. Inhibiting methylation with 5-azadC in FLS treated with TNF-α resulted in upregulation of *PTEN* mRNA and protein levels, downregulation of proinflammatory cytokines (IL-1β, IL-6, and IL-17A) and chemokines (IL-8, CCL-2, CCL-3, and CCL-8), and suppression of FLS activation. The various cytokines form a complex network in RA and exert physiologic effects through overlapping and mutual regulatory functions. Chemokine-induced activation of FLS was shown to aggravate inflammation and bone destruction in RA (You et al., 2014; Jia et al., 2018). Given their abundant expression in the synovium of RA patients, these chemokines may facilitate FLS recruitment and activation (Eljaafari et al., 2012; Angiolilli et al., 2017). In the present work, *PTEN* overexpression in RA FLS treated with TNF-α resulted in the downregulation of proinflammation cytokines and chemokines in FLS. Moreover, intra-articular injection of adenovirus expressing *PTEN* into the knees of AIA model rats markedly reduced inflammatory cell infiltration and paw swelling; this was accompanied by downregulation of IL-6 and TNF-α in the serum and in synovial tissues. IL-6, IL-1α, IL-1β, and keratinocyte-derived chemokine (KC) were shown to be elevated in lung lysates of *PTEN*-null mice (Riquelme et al., 2017). Taken together, our results indicate that altered *PTEN* expression mediated by DNA methylation contributes to the pathogenesis of RA.

This study had some limitations. Firstly, the sample sizes (both clinical samples and AIA rats) were small. Secondly, a *PTEN* knockout mouse and other RA models could have been used to clarify the role of PTEN in RA. Nonetheless, we demonstrated that PTEN plays an important role in RA pathogenesis by modulating the production and secretion of proinflammatory cytokines and chemokines and FLS activation *via* the AKT signaling pathway. We also showed that the expression of PTEN itself in RA is regulated by DNA methylation. These findings suggest that therapeutic strategies targeting *PTEN* methylation may be effective in preventing the development of RA.

## Data Availability

The original contributions presented in the study are included in the article/Supplementary Material, further inquiries can be directed to the corresponding authors.

## Supplementary Material

Supplementary Figure 1 Inhibition of methylation alleviates inflammation in the synovium of rats with AIA. Rats were intraperitoneally injected with 5-azadC (0.7 mg/kg). **(A)** PTEN, TNF-α, and DNMT1 protein levels in synovial tissue detected by western blotting. **(B)** TNF-α and PTEN mRNA levels in synovial tissue detected by qRT-PCR. **(C, D)** IL-1β, IL-6, and IL-17A mRNA **(C)** and protein **(D)** levels in synovial tissue analyzed by qRT-PCR and western blotting, respectively. **(E, F)** IL-1β, IL-6, IL-17A, TNF-α, and PTEN mRNA **(E)** and protein **(F)** levels in synovial tissue analyzed by qRT-PCR and western blotting, respectively. Values represent mean ± SD. ^##^P<0.01 vs. normal group; *P<0.05, **P<0.01 vs. AIA group.
