# Supplementary material for: PTEN Methylation Promotes Inflammation and Activation of Fibroblast-Like Synoviocytes in Rheumatoid Arthritis
Source: Front Pharmacol. 2021 Jul 8;12:700373. doi: 10.3389/fphar.2021.700373 (PMC8296842; doi:10.3389/fphar.2021.700373)
Supplement: Supplementary file 1 [file DataSheet1.doc]

**Supplementary Table 1 The information of RA patients.**

| Group | Number(mean±SD) |
| --- | --- |
| Male | 1 |
| Female | 5 |
| Age | 54.67±10.17 |
| ESR (mm/h) | 42.33±30.86 |
| CRP (mg/L) | 24.32±32.25 |
| RF (IU/ml) | 84.7±94.46 |
| DAS28-ESR | 5.59±0.50 |

**Supplementary Table 2 Methylated and unmethylated PTEN primer sequence.**

| Gene | Forward primer sequences | Reverse primer sequences |
| --- | --- | --- |
| Human |  | |
| PTEN (methylated) | 5'-GATGAGGTGATATACGTTGGCG-3' | 5'-TTTACACCGCTATCGAATCACAAT-3' |
| PTEN (unmethylated) | 5'-GGATGAGGTGATATATGTTGGTGAT-3' | 5'-TTTTACACCACTATCAAATCACAATCA-3' |
| Rat |  | |
| PTEN (methylated) | 5'-CGGTCGGTGTTAAGTTTTTCGT-3' | 5'-AAAACGAATAATCCTCGCAACG-3' |
| PTEN (unmethylated) | 5'-ATTTGGTTGGTGTTAAGTTTTTTGT-3' | 5'-AAAAAACAAATAATCCTCACAACAAAC-3' |

**Supplementary Table 3 Q-PCR primer sequence (Sequence of the primers used for q-PCR experiments)**

| Gene | Forward primer sequences | Reverse primer sequences |
| --- | --- | --- |
| Rat |  | |
| PTEN | 5'-TCCTCCAACTCAGGACCCAC-3' | 5'-TCACCACACACAGGCAATGG-3' |
| IL-1β | 5'-TGACCCATGTGAGCTGAAAG-3' | 5'-AGGGATTTTGTCGTTGCTTG-3' |
| IL-6 | 5'-GAGCCCACCAGGAACGAAAGTC-3' | 5'-TGTTGTGGGTGGTATCCTCTGTGAA-3' |
| TNF-α | 5'-ATGTCTCAGCCTCTTCTCATTC-3' | 5'-GCTTGTCACTCGAATTTTGAGA-3' |
| IL-17A | 5'-TGCCTGATGCTGTTGCTGCTAC-3' | 5'-GGTGAAGTGGAACGGTTGAGGTAG-3' |
| β-actin | 5'-CCCATCTATGAGGGTTACGC-3' | 5'-TTTAATGTCACGCACGATTTC-3' |
| Human |  | |
| PTEN | 5'-TTGCAATCCTCAGTTTGTGG-3' | 5'-AGGTAACGGCTGAGGGAACT-3' |
| IL-1β | 5’-GGACAAGCTGAGGAAGATGC-3’ | 5’-TCGTTATCCCATGTGTCGAA-3’ |
| IL-6 | 5’-CACACAGACAGCCACTCACC-3’ | 5’-AGTGCCTCTTTGCTGCTTTC-3’ |
| IL-17A | 5’-CGGACTGTGATGGTCAACCTGAAC-3’ | 5’-GGTCCTCATTGCGGTGGAGATTC-3’ |
| IL-8 | 5’-TCTCTTGGCAGCCTTCCTGA-3’ | 5’-TTTCTGTGTTGGCGCAGTGT-3’ |
| IL-10 | 5’-TTACCTGGAGGAGGTGATGC-3’ | 5’-GGGAAGAAATCGATGACAGC-3’ |
| MMP-3 | 5’-GGCCAGGGATTAATGGAGAT-3’ | 5’-TGAAAGAGACCCAGGGAGTG-3’ |
| MMP-9 | 5’-GTACCACGGCCAACTACGAC-3’ | 5’-GCCTTGGAAGATGAATGGAA-3’ |
| TIMP-1 | 5’-TGACATCCGGTTCGTCTACA-3’ | 5’-TGATGTGCAAGAGTCCATCC-3’ |
| CCL-2 | 5’-CCTTCATTCCCCAAGGGCTC-3’ | 5’-CTTCTTTGGGACACTTGCTGC-3’ |
| CCL-3 | 5’-TGCAACCAGTTCTCTGCATC-3’ | 5’-TGGCTGCTCGTCTCAAAGTA-3’ |
| CCL-8 | 5’-CTGCGCCAACACAGAAATTA-3’ | 5’-TGAATTCTCAGCCCTCTTCAA-3’ |
| β-actin | 5’-GCCAACACAGTGCTGTCTGG-3’ | 5’-CTCAGGAGGAGCAATGATCTTG-3’ |
