# Supplementary figures and images for: PTEN Methylation Promotes Inflammation and Activation of Fibroblast-Like Synoviocytes in Rheumatoid Arthritis
Source: Front Pharmacol. 2021 Jul 8;12:700373. doi: 10.3389/fphar.2021.700373 (PMC8296842; doi:10.3389/fphar.2021.700373)

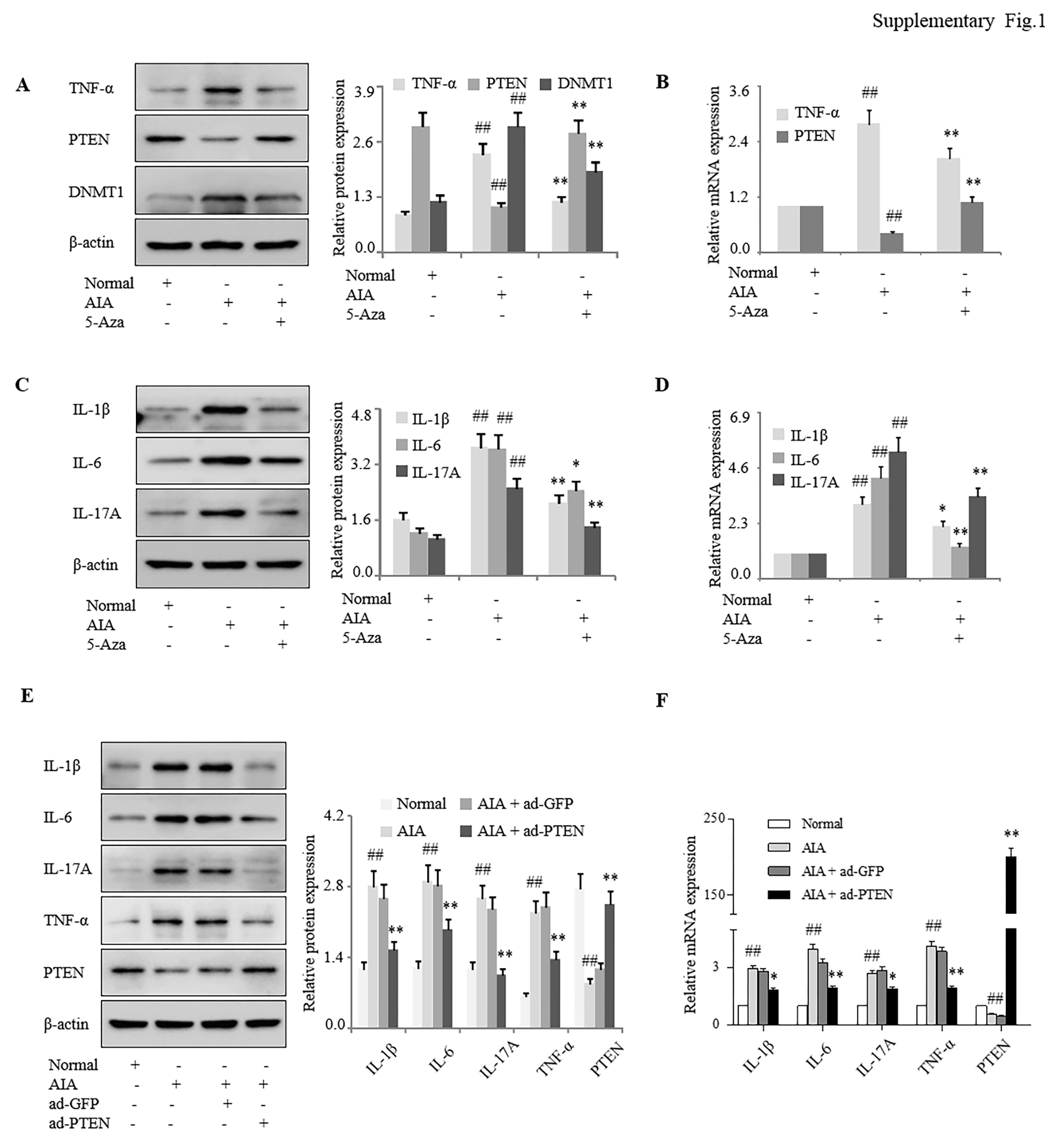

Supplement: Supplementary file 2 [file Image1.TIF]
